# Twelve-Month Survival on Chronic Continuous Milrinone Infusion and Proarrhythmic Complications: A Case Report and Literature Review

**DOI:** 10.7759/cureus.95054

**Published:** 2025-10-21

**Authors:** Sachin Sapkota, Sindhu G. Rajendra, Vishakh Prakash, Vaishnavi Sabesan, Jyothsna Goranti, Sandesh Murali, Tariq Siddiqui

**Affiliations:** 1 Internal Medicine, The Hospitals of Providence - Transmountain/Texas Tech University Health Sciences Center El Paso, El Paso, USA; 2 Invasive-Interventional Cardiology, Center of the Heart - A Providence Medical Partners Practice, El Paso, USA

**Keywords:** advanced heart failure, heart transplant, ionotrope, milrinone, ventricular arrhythmia

## Abstract

Advanced heart failure with reduced ejection fraction remains a major challenge, especially in patients ineligible for transplant or mechanical support. Continuous intravenous milrinone infusion is increasingly used as palliative or bridging therapy, improving cardiac output by enhancing contractility and reducing afterload, with emerging evidence of diastolic benefits; however, it carries a significant proarrhythmic risk.

We present a 68-year-old male patient with advanced heart failure due to mixed cardiomyopathy, status post automatic implantable cardioverter defibrillator (AICD), and decompensated liver cirrhosis who has been on 12 months of outpatient milrinone infusion. Initially started on milrinone as a bridge to transplant, the therapy transitioned to palliation because of ineligibility for cardiac and liver transplantation. The patient showed notable symptomatic improvement, including resolution of recurrent pleural effusions that had previously required thoracentesis every one to two months. Electrocardiogram (EKG) showed occasional premature ventricular contractions (PVCs). Echocardiogram revealed an ejection fraction of 15-20% and grade 3 diastolic dysfunction. During this hospitalization for COVID-19 pneumonia, multiple arrhythmic events were noted, including frequent nonsustained and one sustained ventricular tachycardia, as well as a 19-second ventricular fibrillation episode terminated by an AICD shock. Milrinone was suspected as a key proarrhythmic factor. Additionally, candidemia associated with the chronic peripherally inserted central catheter (PICC) line was identified. After dose titration, arrhythmic episodes decreased significantly. The patient ultimately opted for comfort care and was discharged with hospice services.

This case illustrates the complex nature of milrinone therapy in end-stage heart failure, balancing symptom relief against significant arrhythmic risk. Although studies show improvement in functional class and reduced hospitalizations with continuous inotropic support, survival benefits remain unclear. Our patient’s prolonged 12-month survival exceeds the average reported in the literature. Outpatient milrinone infusion offers meaningful symptomatic relief and possibly extended survival in patients with advanced heart failure. Larger prospective studies are needed to define the true benefit-risk profile and mortality outcomes of chronic inotropic therapy.

## Introduction

Heart failure (HF) has been described as a global pandemic, with an estimated 64.3 million individuals affected worldwide in 2017 [1,2]. According to the 2021 American Heart Association Statistical Update, the prevalence of HF was 6 million, representing approximately 1.8% of the total US population, and is projected to increase to 8 million, accounting for 3% of the total population by 2030 [3]. Despite significant progress in the treatment of HF with reduced ejection fraction (HFrEF), some patients with end-stage disease remain dependent on continuous inotropic support when surgical options are not viable. In such cases, outpatient milrinone infusion is often utilized either as a bridge to transplantation or as palliative therapy [4]. As a phosphodiesterase-3 inhibitor, milrinone enhances cardiac output by increasing contractility, reducing afterload, and improving ventricular relaxation, thereby alleviating symptoms and optimizing hemodynamics [5]. Additionally, growing evidence suggests that milrinone contributes to diastolic function by enhancing ventricular compliance and lowering filling pressures [6]. However, its prolonged use is not without risks. One of the most concerning complications is its proarrhythmic potential, which can trigger dangerous ventricular arrhythmias and even sudden cardiac death. This risk is supported by the two largest prospective trials: OPTIME-CHF (Outcomes of a Prospective Trial of Intravenous Milrinone for Exacerbations of Chronic Heart Failure), which reported a 28% higher mortality in the milrinone group, and PROMISE (Prospective Multicenter Imaging Study for Evaluation of Chest Pain), which demonstrated increased rates of hypotension and atrial arrhythmias in patients receiving milrinone [4,7,8]. While milrinone offers symptomatic relief and improved circulation, its long-term safety remains a critical concern in the management of advanced HF.

We present a unique case of a patient with advanced systolic and diastolic HF who survived for 12 months on continuous milrinone infusion, with significant improvement in symptom burden. With an implantable cardioverter defibrillator (ICD) in place, our patient exceeded the average survival reported in the literature [9-12], demonstrating a potential survival benefit in the ongoing debate regarding the use of continuous intravenous inotropes.

## Case presentation

A 68-year-old man with a past medical history of advanced systolic and diastolic heart failure secondary to mixed ischemic and non-ischemic cardiomyopathy on continuous milrinone drip, status post biventricular automatic ICD (AICD), coronary artery disease (CAD) status post stent ×3, peripheral artery disease (PAD), decompensated liver cirrhosis, hypertension, and diabetes mellitus type 2 presented to our facility with the complaint of cough and leg swelling that had progressively worsened recently. He was referred from urgent care after being diagnosed with COVID-19 pneumonia. The patient had been on a continuous milrinone infusion at 0.375 mcg/kg/min at home for 12 months. The patient expressed significant improvement in symptoms after milrinone initiation, no longer requiring thoracentesis, which was previously necessary every month. He was initially started on a milrinone drip as bridging therapy, being approved for heart transplantation but denied liver transplantation due to frailty and comorbidities. The patient was not on optimal guideline-directed medical therapy as he could not tolerate it due to low blood pressure. Home medications included metoprolol (12.5 mg daily), torsemide (40 mg daily), spironolactone (100 mg daily), and continuous milrinone infusion (0.375 mcg/kg/min).

**Figure 1:**
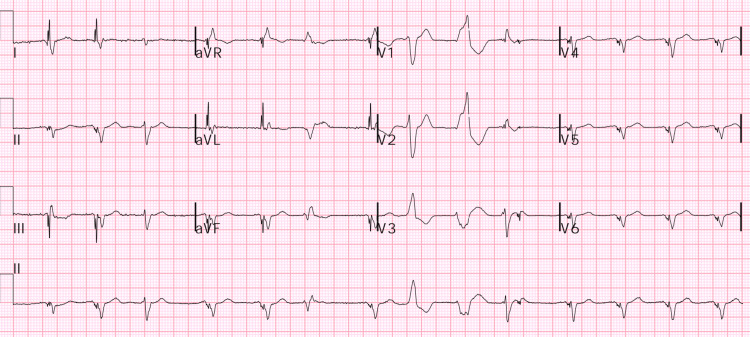
Electrocardiogram (EKG) on admission EKG on admission shows paced rhythm with occasional paroxysmal ventricular contraction (PVCs).

**Figure 2:**
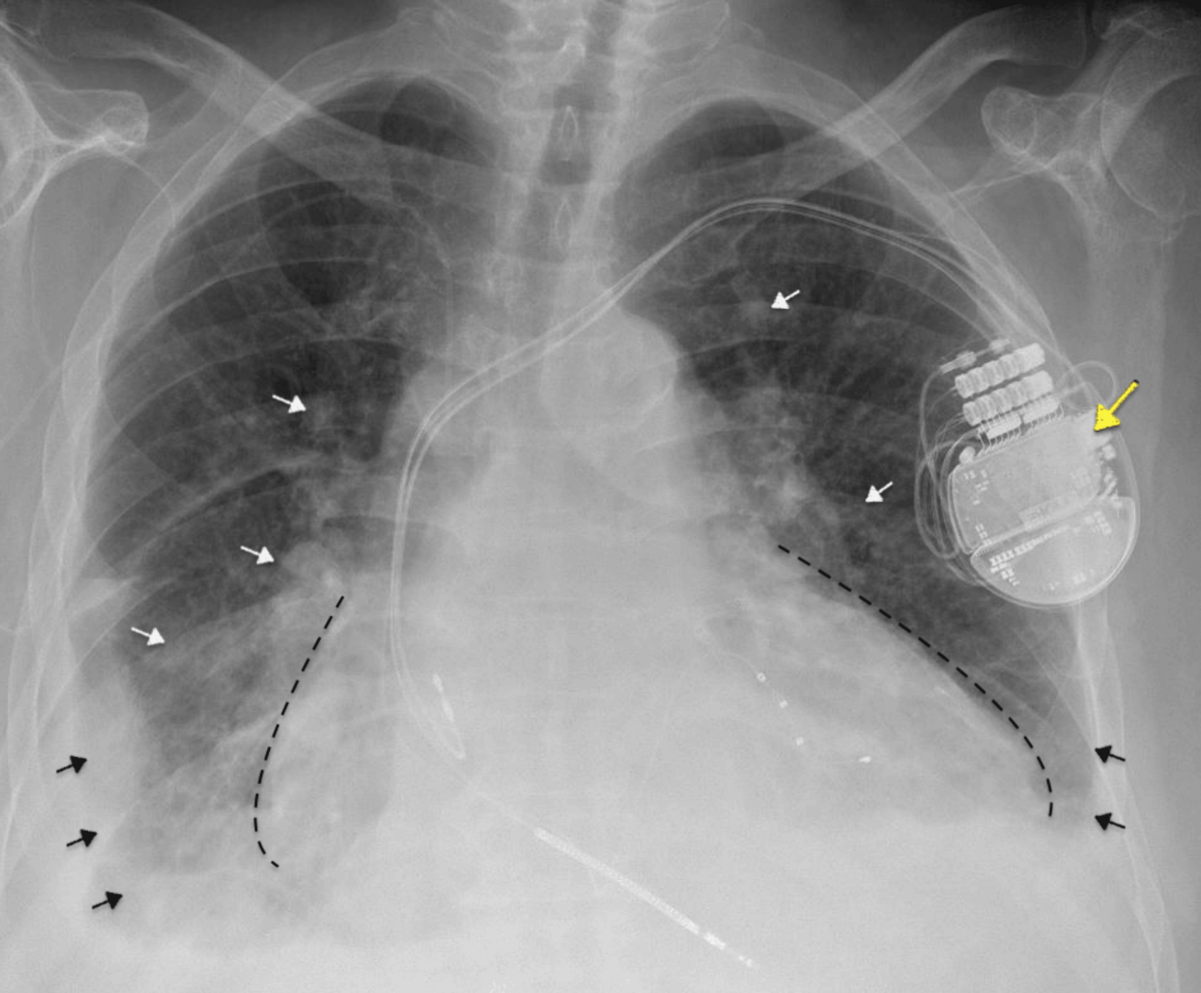
Posteroanterior chest X-ray (CXR) on admission Chest X-ray demonstrates bilateral pleural effusions (black arrows), more prominent on the right, along with patchy airspace opacities in the lung bases (white arrows) (right greater than left) consistent with pulmonary edema, and an enlarged cardiac silhouette indicating cardiomegaly (black dotted lines). Automatic implantable cardioverter defibrillator (AICD) in place (yellow arrow).

**Figure 3:**
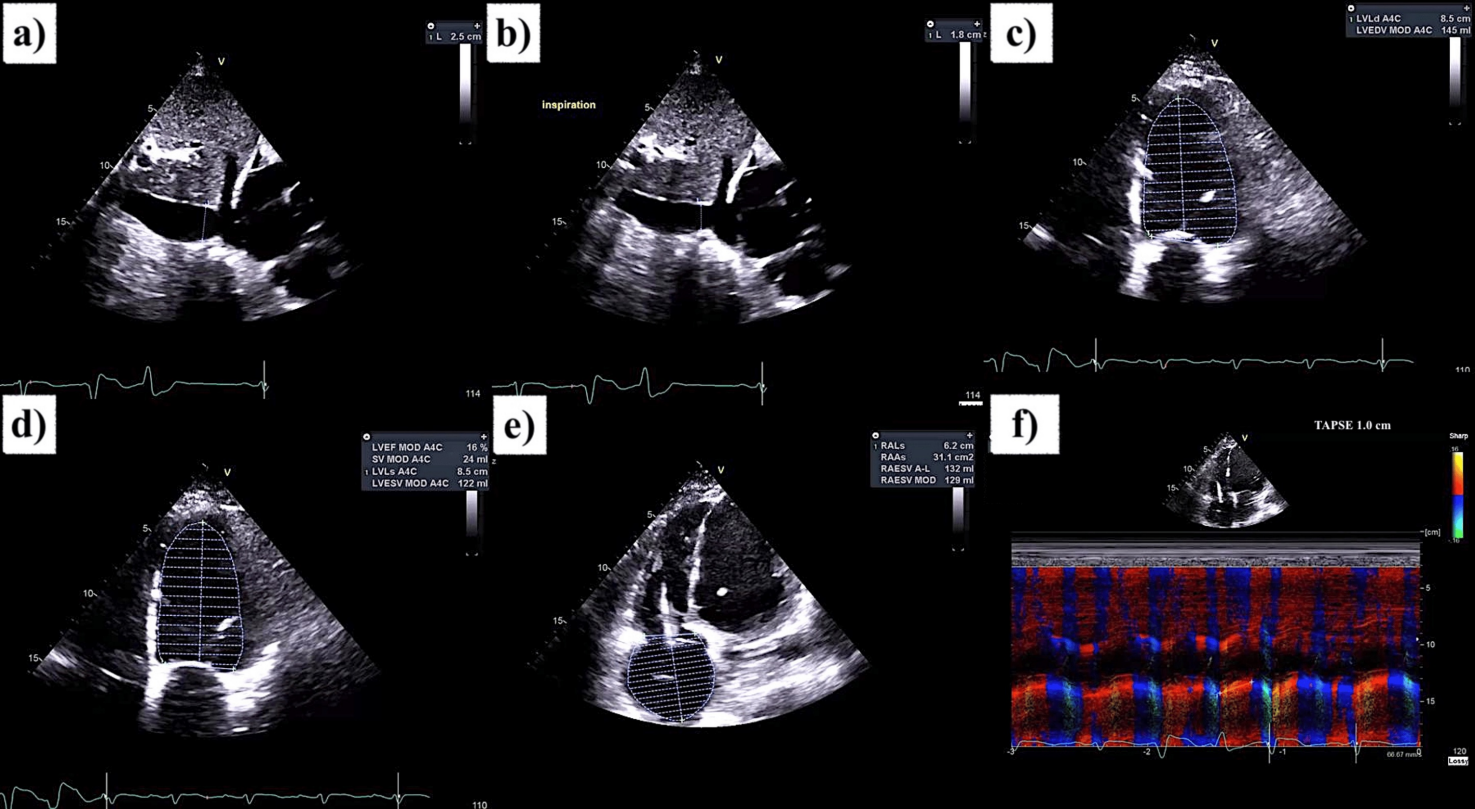
Echocardiogram on admission demonstrating severe global systolic and diastolic dysfunction (a and b) IVC diameter on expiration (2.5 cm) and inspiration (1.8 cm), respectively (<50% collapse on inspiration), indicating elevated RAP and severe volume overload. (c and d) LV size in diastole (145 mL) and systole (122 mL), respectively, indicating a severely dilated left ventricle, global LV hypokinesis, and severely reduced LV ejection fraction (16%). (e) Severely dilated right atrium (RA) with an area of 31.1 cm². (f) Color Doppler echocardiogram showing reduced tricuspid annular plane systolic excursion (TAPSE, 1.0 cm), indicating severely reduced right ventricular (RV) systolic function. IVC: inferior vena cava; RAP: right atrial pressure; LV: left ventricle; RA: right atrium; TAPSE: tricuspid annular plane systolic excursion; RV: right ventricle; EF: ejection fraction.

On initial evaluation, the patient had mild to moderate respiratory distress, tachycardia (HR 112), hypotension (90s/60s), and required 2 L O₂ to maintain saturation. Examination revealed elevated JVP, 3+ pitting edema of both lower extremities, and bilateral basal crackles on lung auscultation. Cardiac examination revealed a severely displaced point of maximal impulse and a systolic murmur in the tricuspid area. EKG showed a paced rhythm with occasional PVCs (Figure 1). The results of laboratory evaluation on admission are shown in Table 1, with significantly elevated BNP and bilirubin. Chest X-ray demonstrated bilateral pleural effusions, more prominent on the right, along with patchy airspace opacities in the lung bases-right greater than left-consistent with pulmonary edema and an enlarged cardiac silhouette (Figure 2). Echocardiogram showed dilated cardiomyopathy with severely reduced LV systolic function (LVEF 15-20%), grade 3 diastolic dysfunction, biventricular and biatrial dilation, moderate to severe tricuspid regurgitation, and severe pulmonary hypertension (Figure 3). The prior echocardiogram a year ago also showed an LVEF of 15-20%.

**Table 1:** Initial laboratory reports on admission and subsequent days of hospitalization until discharge WBC: white blood cell count, HCO₃: bicarbonate, ALT: alanine aminotransferase, AST: aspartate aminotransferase, BNP: B-type natriuretic peptide, COVID-19 PCR: coronavirus disease 2019 polymerase chain reaction test, IU: international unit.

Laboratory Test	Reference Range	Day 1 (Admission)	Day 4	Day 10	Day 17 (Discharge)
WBC (10³/mm³)	4.5–11	9.67	9.36	7.38	12.23
Hemoglobin (g/dL)	12–16	15.5	15.4	13.4	14.9
Platelet (10³/mm³)	130–400	210	161	201	251
Sodium (mmol/L)	135–145	121	129	124	119
Potassium (mmol/L)	3.5–5.1	5.0	4.0	4.8	4.4
HCO₃ (mmol/L)	21–31	23	29	32	31
Creatinine (mg/dL)	0.3–1.3	1.3	1.3	1.2	1.3
ALT (IU/L)	0–40	11	-	-	14
AST (IU/L)	0–40	17	-	-	27
Total bilirubin (mg/dL)	0–1.0	2.2	-	-	1.0
BNP (pg/mL)	≤100	1924	1156	-	-
COVID-19 PCR		Detected	-	-	-

**Figure 4:**
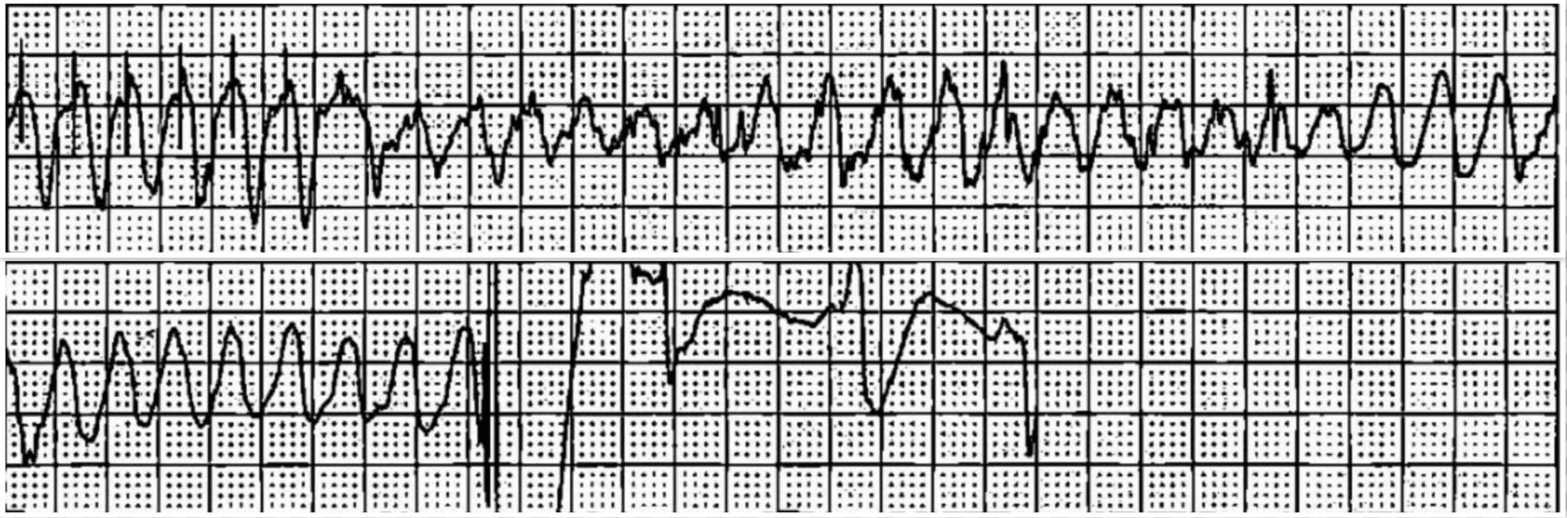
Telemetry recording during hospitalization showing episode of ventricular fibrillation Telemetry recording showing a 19-second episode of ventricular fibrillation, which was terminated by the automatic implantable cardioverter-defibrillator (AICD), successfully converting the rhythm to sinus rhythm with intermittent pacing.

Considering his comorbidities and chronic milrinone infusion for advanced HF, he was admitted to the Coronary Care Unit. The patient was started on torsemide 50 mg twice daily (orally) for diuresis, as he was severely volume overloaded, followed by once-daily dosing from day 2. Aggressive diuresis was limited due to hypotension. Milrinone infusion was continued at his home dose of 0.375 mcg/kg/min. Digoxin 0.25 mg daily (orally) and metoprolol 12.5 mg twice daily (orally) were also started, but metoprolol was later discontinued due to low blood pressure. At the same time, the patient was also being treated for community-acquired pneumonia, hypervolemic hyponatremia, and candidemia as a complication of the long-term PICC line used for milrinone infusion. On the third day of admission, the patient had an episode of asymptomatic non-sustained ventricular tachycardia. The situation became more concerning when telemetry began recording multiple and frequent proarrhythmic events. The digoxin dose was reduced to 0.125 mg daily (orally). The patient later had one episode of sustained ventricular tachycardia and one 19-second episode of ventricular fibrillation (Figure 4), which reverted to sinus rhythm after an AICD shock. Multiple episodes of NSVT and PVCs continued during the hospital stay. He was started on an amiodarone drip (1 mg/min for 6 hours, then 0.5 mg/min).

Though he had multiple risk factors for developing arrhythmic events, milrinone appeared to be the main factor. The cardiologist decided to titrate down the milrinone dose to 0.25 mcg/kg/min and then to 0.125 mcg/kg/min, as the patient tolerated the dose reduction well. The patient continued to have frequent PVCs and two additional episodes of asymptomatic NSVT but did not experience any sustained VT/VF during the last eight days of hospitalization. The frequency of arrhythmic events was reduced significantly with milrinone titration. On further inquiry, the patient mentioned that he had started experiencing multiple episodes of dizziness and syncope after initiation of the milrinone drip, which could be related to its proarrhythmic effect, though ICD interrogation was not performed during this hospitalization. During his stay, the transplant team evaluated the patient for a second opinion and advised that he was not a suitable candidate for cardiac or liver transplantation. Goals of care were discussed with the patient, and he opted for comfort care. Active medications, including the milrinone drip, were discontinued, and the AICD was deactivated. The patient was discharged with home hospice care.

## Discussion

For patients with refractory advanced HF and NYHA class IV symptoms despite optimal guideline-directed medical therapy, treatment options are limited, particularly for those who are not suitable candidates for ventricular assist devices or heart transplantation. Due to the scarcity of donor organs, only a small number of advanced HF patients are eligible for heart transplantation [13,14]. While more patients may be candidates for alternative surgical options, factors such as advanced age, frailty, or non-cardiac comorbidities may preclude eligibility for left ventricular assist device (LVAD) implantation [14]. As a result, an increasing number of patients are receiving long-term intravenous inotropic therapy to alleviate symptoms [5,14].

Intravenous inotropes have been commonly used for short-term management of cardiogenic shock [4,15,16]. There is growing clinical use of long-term milrinone for palliative purposes. The 2022 AHA/ACC (American Heart Association and the American College of Cardiology) guidelines list short-term IV inotropes for the management of cardiogenic shock as a Class I indication and continuous long-term IV inotropes as a bridge to mechanical circulatory support or cardiac transplantation, or for palliation in those not eligible for either, as a Class II indication [4]. Of the FDA-approved, commercially available options, dobutamine and milrinone are the most commonly used continuous inotropes for palliative therapy [14].

Milrinone inhibits phosphodiesterase-3 (PDE-3), preventing the breakdown of cAMP (cyclic adenosine monophosphate), which increases calcium influx into myocytes and enhances myocardial inotropy. While there is no strong evidence indicating that one inotrope is superior to another, milrinone’s ability to lower pulmonary artery pressures makes it a promising therapeutic option for patients with pulmonary hypertension and/or right-sided HF [5]. Although the initial PROMISE trial demonstrated that long-term therapy with oral milrinone increased morbidity and mortality in patients with severe chronic HF, suggesting against its use [8], a double-blind pilot study reported outpatient infusion of inotropes to be safe and effective in improving HF symptoms, reducing hospitalizations and emergency room visits, and significantly enhancing six-minute walk test scores compared with placebo [17]. Milrinone also appears to provide these benefits more quickly than dobutamine [17]. A systematic review and meta-analysis by Nizamic et al. demonstrated an average improvement of 1.2 NYHA (New York Heart Association) functional class and reduced symptom burden without affecting overall survival among patients receiving chronic intravenous inotropic support for advanced HF [18].

Over the decades, with accumulating evidence, the ACC/AHA has updated its guidelines to recommend intravenous milrinone use for palliation in refractory advanced HF [4,17,18]. However, inotropes used for palliative care carry risks of arrhythmias and catheter-related infections, as observed in our patient. In a study by Acharya et al., 17% of patients experienced one or more ICD shocks during follow-up, and 29% had one or more infections [19]. Although ICDs can reduce arrhythmia-related mortality, their continued use should be discussed with patients who may choose to deactivate them for palliative purposes or in the setting of frequent shocks [4,19,20].

A retrospective cohort study by Rao et al. found that patients receiving continuous intravenous inotropic support (CIIS) as palliative therapy had an average survival of 6.2 months after starting treatment [10]. Those on CIIS as bridge therapy who did not undergo surgical intervention had an average survival of 8.6 months [10]. Similarly, Eaton et al. [9] and Cole et al. [11] reported average survival durations of 7.6 and 5.4 months, respectively. Our patient survived for 12 months on bridging intravenous milrinone therapy, with improvement in symptoms despite multiple arrhythmic events and ICD shocks. He experienced significant symptomatic improvement and a survival duration longer than the average reported in the current literature.

## Conclusions

This case highlights the potential role of continuous intravenous milrinone support as both a bridging and palliative strategy, demonstrating symptomatic improvement and prolonged survival beyond reported averages, despite recurrent arrhythmic events. While morbidity benefit is evident, mortality benefit cannot be concluded from a single case. Despite the growing use of CIIS, evidence supporting its effect on patient-related outcomes for outpatient infusion therapy remains limited. Milrinone and dobutamine have shown promising effects in improving function and symptoms, though without demonstrated survival benefit. However, the current literature on CIIS has notable limitations. There is a clear need for prospective, placebo-controlled trials to better define the impact of CIIS on survival, as well as its potential harms and benefits.
